# General Observations on Pathology

**Published:** 1826-03

**Authors:** Wm. Stoker

**Affiliations:** senior Physician to the Fever Hospital and House of Recovery, Cork-street, Dublin.


THE LONDON
### Medical and Physical Journal.

No. 3 OF VOL. LV.] MARCH, 1826. [No. 325.

For many fortunate discoveries in medicine, and for the detection of numerous errors, the world is indebted to the rapid circulation of Monthly Journals; and there never existed any work, to which the Faculty, in Europe and America, were under deeper obligations, than to the Medical and Physical Journal of London, now forming a long, but an invaluable, series.—RUSH.

### ORIGINAL COMMUNICATIONS,
### SELECT OBSERVATIONS, &c.

ART. I.—*General Observations on Pathology.* By WM. STOKER, M.D. senior Physician to the Fever Hospital and House of Recovery, Cork-street, Dublin.

PREVIOUS to the further illustration of the pathological principles* adopted in my work on Dropsy, which I intend to deduce from the intimate correspondence between the intensity of the symptoms and that of the disorder of the animal fluids, in the aggravated forms of epidemic prevailing in this country for the last three years, I am desirous to review the evidence I have already offered in support of these principles, inasmuch as I am very apprehensive that, without such a retrospect, the facts which my opportunities enable me to bring forward on a question which I think deeply interesting, both to physicians and their patients, would not receive that patient and due consideration which they deserve. And besides, the observations I have to bring forward merit attention chiefly (as it appears to me,) from their connexion and accordance with those arguments and facts on which I had first promulgated my pathological opinions.

Whilst, therefore, I wait the close of *this*, which may be but too truly entitled "*læthiferus annus*," in order to complete those numerical statements which I am preparing in the concise form of Tables for the years 1823, 24, and 25, I beg leave, as briefly as I can, to offer, with a view to such a retrospect, some observations on the origin of the exclusive pathology of the solidists, as now inculcated in the schools of Physic,—on the imperfections necessarily attached to such a system,—and, lastly, on the failure of its partisans, especially as respects the

* I regret that my efforts to establish these principles have been sometimes mistaken for attempts to restore the old humoral pathology; and, therefore, think it necessary to add, that I should deem the exclusive pathology of the fluids necessarily as imperfect as that founded solely on the solids.

pathology of fever, to form a correct *rationale* as to the nature or treatment of that disease.

It would be necessary to go back to very early periods in the history of medicine, to trace the errors of this exclusive system from their origin, and to examine the causes of the various revolutions this science has undergone in successive ages,—causes which, I think, would be found not so intimately connected with want of zeal or talents in any of the methodical, mechanical, chemical, or anatomical sects of physicians, as with the dispositions of each to advocate their theories by their own creeds exclusively, and to detect with microscopic eye the fallacies or fictions of those which differ from them. So extensive an inquiry, however, would be incompatible with the limits to which I should here confine myself; and, as I can refer to the works of the learned Cabanis, which are very full on that subject, I may commence from the period when Cullen, Brown, and Darwin, by the aid of their extraordinary abilities, and the influence on medical opinions which their high characters had, transferred to Great Britain the doctrines of solidism, which had been previously promulgated on the continent by the eloquence of Stahl, Hoffman, and Baglivi.

Influenced by the dogmas of these distinguished British physicians, medical writers and lecturers thenceforward framed all their theories, (but fortunately not entirely their practice,) taking little or no account of the state of the fluids in disease; notwithstanding that Sydenham's practical observations, as well as Boerhaave's more theoretic writings, had till that time taught that the condition of the blood, and of its derivative fluids, in disease, should form necessary parts of pathology.

The new theorists were too acute observers not to perceive that the destruction of previous systems, at least so rapidly, was owing to their not bearing the test of the analytical method, which had been employed so effectually for the detection of error in the other sciences: they gave theirs, therefore, as much as possible, the semblance of being founded according to inductive philosophy. In their explanation of the phenomena of diseases, no particular notice of the condition of either solids or fluids was taken, and presented little more than a brief exposition of the principal symptoms, ingeniously interwoven with either an imaginary hypothesis of a proximate cause or essence of disease, dependent either on a transient condition of the solids, which could not be submitted to subsequent investigation, or connected with abstruse ideas of the laws of the animal economy, which the metaphysicians of that day pretended to have discovered, and by them vainly attempted to disclose the occult and ultimate relations between the operations of mind and body; and, assuming them as data, employed arguments which would lead to materialism in the moral, as certainly as to solidism in the physical, world. But, however well contrived these fictions might be for deception, they soon sunk into well-merited contempt, under impartial and vigorous scrutiny.

But, though that system of pathology now known by the name of Solidism was first introduced into England, and is still to a certain degree supported there on such speculations, it was in the founders of the great Anatomical School at Windmill-street that it was sustained by its ablest and warmest advocates, who, conscious of their extensive powers, derived from their anatomical skill in post-mortem examinations, laboured with unwearied industry to form a system solely from the facts collected in examining the alterations of structure produced by diseases; a task recommended to their ardent minds, not only by attachment to their favourite pursuit, but also on account of its being opposed in principle to those untenable parts of the theories of the humoral pathologists, which they had been successfully employed in exposing.

The immense array of facts which the distinguished brothers, William and John Hunter, made for this purpose, during their widely extended inquiries in dissection, recorded in their literary works, as well as in their museums, has (independent of the great depositories since added by their pupils and followers,) been justly the theme of admiration, as well for the persevering assiduity which it evinces, as for the importance which has been attached to it, or the influence which it has ever since had on those making similar collections, or on the improvements in medicine, but more particularly in surgery. Indeed, without meaning any conceit unsuitable to so solemn a subject, it may be said of the deathless name they have laboured so effectually to establish, that, however "the evil they did might live after them, the good was not interred with their bones;" for there is scarcely any civilised country which does not at present possess either copies of their works or models of their museums. Fortunately it is only necessary to refer thus generally to these works, and I shall confine my observations to that faithful epitome of them entitled the "Morbid Anatomy," the text-book of dissectors since its publication; a work with which the well-earned fame of the late lamented Dr. M. Baillie is intimately connected, and which was no less indebted for its celebrity to its author's highly respected name. In it the views of his distinguished relatives in collecting preparations during the dissection of morbid parts, to which he was, even during their life-time, an active and large contributor, are zealously and ably extended by all that his own extensive opportunities, as lecturer in the great school of Anatomy, presented in favour of similar principles. It was composed, indeed, with the avowed object of explaining, "more minutely than had hitherto been done, the changes of structure arising from morbid actions in some of the most important parts of the human body;" and, I might say, never would have led to the errors which others have committed under the affectation of having its sanction, if the subsequent attempts to found a purely solid pathology had been guided by the caution with which he advanced, and the faithfulness with which he delineated, those boundaries he had reached; or if, like him, they had candidly defined their objects for extending them.

As an illustration of this opinion, and an apology for the freedom with which I shall venture to discuss some subsequent parts of this work, I am induced to take the following extracts from its Preface:—"The human mind is prone to form opinions upon every subject which is presented to it, but, from natural indolence, is frequently averse to inquire into the circumstances which can alone form a sufficient ground for them. When, however, the mind shall be obliged to observe facts which cannot be reconciled with such opinions, it will be evident that such opinions are ill-founded, and they will be laid aside." And afterwards he says, "that anatomy may be said to have arrived at a high pitch of perfection; but our knowledge of structure produced by disease, which may be called morbid anatomy, is still very imperfect."*

Now, if the entire of the work be examined by the tests suggested in these extracts from its Preface, I feel well assured that the most accomplished anatomist would be most ready to acknowledge that, as a system of pathology, it is imperfect, even in the diseases treated of; for, in many cases similar to these, (especially such as are strictly medical,) the appearances stated to have been found on dissection, by which the previous symptoms were supposed to be accounted for, have been observed when no such corresponding symptom previously occurred. The converse of this is also true; for, when dissection presented no alteration of structure, such symptoms have preceded.

The frequency of such coincidences as those pointed out in the Morbid Anatomy, have no doubt afforded material assistance in diagnostics and prognostics; and, perhaps, it is on those points only that pathological information can be effectually promoted by such inquiries. Hitherto, at least, the views they afford do not extend beyond effects that may supervene, either soon after the commencement or shortly before the fatal termination of diseases, and therefore cannot, as the essential characteristics, guide the physician in prescribing—

* *Morbid Anatomy*, &c. &c. by M. BAILLIE, M.D. F.R.S. &c.—London, 1812.

"Officium medici circa ægrum hominem est sanare."

I might quote numerous cases and dissections to support the foregoing assertions, but my limits necessarily confine me to reference; and I am persuaded that, if those which may be found in this and other periodical Journals, commencing with meningeal apoplexy, and proceeding with the affections of the viscera in all the other cavities, be impartially analysed, they will be sufficient to ensure a rigorous examination of those details which will constitute the additional evidence I have promised; and which is the most favourable reception I wish for them.

As the opinions of the author of the work we have been just considering, respecting those preternatural appearances which the blood drawn in certain diseases evinces must necessarily have influenced his notions of pathology, as well as those of all others who adopted them, they should not be passed unnoticed here; and I may confess additional gratification in referring to those experiments* which I instituted to prove their fallacy, since they have by some been considered decisive;† being fully convinced that, if I could thus succeed in abolishing this false principle of the anatomical sect of pathologists, which BACON would have ranked amongst the "*idola tribus*," greater freedom to the investigation of truth would be the consequence, and facts more fairly appreciated. Those alterations from the natural condition of the blood, as indicated by the preternatural appearances it presents when drawn, could not fail to be esteemed as sufficient causes for the interruption of health, and adequate explanation of the symptoms of disease; as much so, surely, as those alterations in structure found after death, on which so much attention has of late been so beneficially bestowed. Perhaps, there is no circumstance more remarkable in the history of science, than that John Hunter, who had attributed, and almost proved, the paramount importance of the blood to life and health, should have rejected the opinion that morbid alterations in it must be succeeded by disease or death, so as to be now quoted as the successful champion of solidism, who had totally exploded the humoral pathology.

The various appearances of the blood drawn in disease, being no longer attributable to any process subsequent to extravasation or death, I have proposed the following explanation: that one kind of the sizy surfaces, or buffy coats, is produced from disordered chyle, (the consequence of unwholesome aliment or impeded digestion,) not being duly prepared for the changes it

---

* See *Pathological Observations*, Part I. pages 37—40.

† See *Medico-Chirurgical Review*, June 1824; also *Quarterly Journal of Medical Science*, London, 1825.

should undergo in the minor circulation, the functions of which it disturbs, and then passes into the sanguiferous system, to be the source of morbid actions. Another kind arises from the morbid condition of the venous blood, in its return from the greater circulation by the vena portæ, unfitting it for the preparatory process which, in health, takes place in its passage through the liver, in order to qualify it to be duly affected by the functions of perfect sanguification, by which it was to be again refitted, in the pulmonary system, for the vital purposes of the greater circulation. Explanations which appear to be further supported by the experiments on the blood instituted since mine, in London and Paris; by which I am further led to hope that they will hereafter be found of practical utility, both in the *ratio symptomatum* and *methodus medendi* of diseases.

The explication given by Dr. Scudamore of such sizy appearances of the blood, I believe also applicable in many instances; for it is very probable that, when the state of the solids is such that they cannot receive the entire quantity of fibrine conveyed to them by the extreme arteries, it accumulates in the sanguiferous system, and is extraneous to the venous system, by which it is returned to the heart.

The various appearances which the blood presents in idiopathic and typhoid fevers, probably arise from changes effected on the vital fluid, by morbific matter supplied to the lacteal or lymphatic system by the absorbents, which, entering by the thoracic duct, I suppose has the same sedative influence which poisons have on the pulmonary and sanguiferous systems. This view of the subject may also assist in explaining the length of time, or latent period, between the application of contagion or infection, and the commencement of febrile paroxysms or morbid actions.

I hope, in the second part of my Pathological Observations, when I am able to bring them out, to enter upon the chemical properties of morbid blood, as it appears when drawn in both idiopathic and typhoid fevers, by which I am persuaded the further investigation of this subject will be promoted, but for which I had neither leisure nor sufficient knowledge of practical chemistry, when preparing the former part; but now hope to have it undertaken by one on whose skill and accuracy in chemical analysis I can fully rely. It may be said, indeed, that such considerations are more strictly physiological, and do not involve the facts of altered conditions of the blood, and their necessary effects in disease, which are most important in a pathological point of view.

The foregoing observations on the origin of the exclusive pathology of the solidists, and of its imperfections, respect medical diseases in general; and, before I proceed to remark on the more recent failures of the partisans of that system, in which I intend to confine myself to the pathology of fever in particular, I shall take the opportunity of explaining a passage in the valuable Digest of the Study of Medicine, by Dr. J. M. Good, lately published, which I shall transcribe for that purpose, viz.

"The most triumphant fact in favour of the Boerhavean hypothesis is, that the crust on the blood in inflammations, and cauma or inflammatory fevers, is often found peculiarly dense. But, as fevers (and certainly the greater number,) are found without any such crust, and as a similar crust, though perhaps not quite so dense, exists under other and very different states of body, as in pregnancy and scurvy *(porphyra)*, even this leading appeal has long lost its power of conviction; whilst the abruptness with which fevers make their assault from sudden occasional causes, and in constitutions of every diversity, forbids the supposition that in such cases a lentor or sizy crasis of the blood, and especially a *glutinosum spontaneum*, can have time to be produced, however, as it may exist occasionally, and be perhaps the source of other disorders. The subject, however, has of late been again taken up by Dr. Stoker, of Dublin, with a view of reviving the humoral pathology in its more important doctrines, and of extending the arguments which have hitherto been urged in its favour."

Thus called upon, I object, "*toto cœlo*," to that part of the Boerhavean doctrines which attributes fever in general to the sizy condition of the blood, which it may be seen is the tenor of my works on that subject; and, so far from such sizy or buffy coats being the necessary attendants on fever, I questioned them even as proofs of inflammation, because they are found in dropsy and other diseases, where neither heat, pain, redness, or quick pulse, (the ordinary signs of inflammation,) preceded. In typhoid fevers, a condition of the blood, the very converse of the morbid one thus indicated, takes place, when the coagulum of the vital fluid is generally broken down, and its serum is of a dark, often of a greenish hue; and, in symptomatic fevers, the buffy coat, I think, generally indicates disturbed sanguification, which is sometimes the effect merely of inflammation. I should further observe, that I have never seen sudden solution of disease in cases where the buffy coat has been deep or dense; such as, Dr. Good very justly states, frequently succeeds slight hemorrhages from the nose or other parts.

My remaining observations on the failure of the partisans of solidism in the pathology of fevers, will consist chiefly of a narration of facts, justly styled the philosophy of history, which have passed under my notice in the great Fever Institution in Cork-street, during twenty-three years that I have been physician to it. By comparing the doctrines which would place idiopathic fevers amongst the phlegmasia of CULLEN, manifestly the fruits of those metaphysical and anatomical theories, which I have already endeavoured to show were branches from the original stock of solidism, I shall state the process by which I examine their validity; but, as I have already, I fear, transgressed the limits for one insertion in this Journal, I shall reserve these remaining observations as a fit programme for the details of the epidemic, on which I intend next to enter.

*York-street; December* 8, 1825.

